# Discovery of circulating proteins associated to knee radiographic osteoarthritis

**DOI:** 10.1038/s41598-017-00195-8

**Published:** 2017-03-09

**Authors:** Lucía Lourido, Burcu Ayoglu, Juan Fernández-Tajes, Natividad Oreiro, Frauke Henjes, Cecilia Hellström, Jochen M. Schwenk, Cristina Ruiz-Romero, Peter Nilsson, Francisco J. Blanco

**Affiliations:** 1 Rheumatology Division, ProteoRed/ISCIII Proteomics Group, INIBIC – Hospital Universitario de A Coruña, A Coruña, Spain; 2 Affinity Proteomics, SciLifeLab, School of Biotechnology, KTH - Royal Institute of Technology, Stockholm, Sweden; 3 Wellcome Trust Centre For Human Genetics, McCarthy’s Group, University of Oxford, Roosevelt Drive, Oxford, OX3 7BN UK; 4 CIBER-BBN Instituto de Salud Carlos III, INIBIC-CHUAC, A Coruña, Spain; 5 RIER-RED de Inflamación y Enfermedades Reumáticas, INIBIC-CHUAC, A Coruña, Spain

## Abstract

Currently there are no sufficiently sensitive biomarkers able to reflect changes in joint remodelling during osteoarthritis (OA). In this work, we took an affinity proteomic approach to profile serum samples for proteins that could serve as indicators for the diagnosis of radiographic knee OA. Antibody suspension bead arrays were applied to analyze serum samples from patients with OA (n = 273), control subjects (n = 76) and patients with rheumatoid arthritis (RA, n = 244). For verification, a focused bead array was built and applied to an independent set of serum samples from patients with OA (n = 188), control individuals (n = 83) and RA (n = 168) patients. A linear regression analysis adjusting for sex, age and body mass index (BMI) revealed that three proteins were significantly elevated (P < 0.05) in serum from OA patients compared to controls: C3, ITIH1 and S100A6. A panel consisting of these three proteins had an area under the curve of 0.82 for the classification of OA and control samples. Moreover, C3 and ITIH1 levels were also found to be significantly elevated (P < 0.05) in OA patients compared to RA patients. Upon validation in additional study sets, the alterations of these three candidate serum biomarker proteins could support the diagnosis of radiographic knee OA.

## Introduction

Osteoarthritis (OA) is the most common rheumatic disease of the developed world and it is increasingly important in current ageing populations, leading to patient chronic disability^1–3^. This disease manifests not only by cartilage degradation but also as an alteration of the whole joint structure, with progressive synovial inflammation and changes on the subchondral bone and osteophyte formation^4^.

Currently, OA diagnosis is mainly symptomatic, resting on the description of pain symptoms and stiffness of the affected joints, the examination of functional capacity based on Western Ontario and McMaster Universities Osteoarthritis Index (WOMAC)^5^, and the evaluation of cartilage radiography^6^ or magnetic resonance imaging (MRI)^7^. However, the sensitivity of radiography is not adequate for detecting small changes, thus when radiographic diagnosis is established, significant joint damage has often already occurred^8–10^. In contrast, MRI is a quite sensible technique and it has been developed for the evaluation of cartilage damage in OA, but it is very expensive and requires a large instrumentation time, which limits its applicability^8, 11^. Moreover, OA has little efficient therapeutics, probably as a consequence of the lack of early diagnosis strategies and techniques for its precise monitoring.

In the last years, biochemical biomarkers have emerged as promising tools in OA diagnosis, with more sensitivity and reliability than plain radiography to detect joint changes that occur in OA^12^. Such markers of osteoarthritis could facilitate early diagnosis of joint destruction, disease prognosis and progression monitoring, which could be detectable with an early biochemical test^13^. Over the years, a series of markers have been proposed that may reflect the synthesis or degradation of the joint tissues. However, despite the active research in this field, currently no single marker is sufficiently validated for its use in OA diagnosis^14–16^. This is mainly due to the lack of validation studies in large populations, which would strengthen the findings to be considered as robust biomarkers for OA^17^.

In the present study, 1032 serum samples from OA patients, healthy control subjects and disease control samples from patients with rheumatoid arthritis (RA) were analysed using a high-throughput affinity proteomic approach based on antibody suspension bead arrays, with the potential to screen hundreds of proteins in hundreds of body fluid samples in parallel^18^. Here, we aimed to identify a panel of serum proteins able to discriminate knee radiographic OA patients from healthy controls. The specificity of the proteins found was evaluated by screening the protein profiles of RA patients.

## Results

### Initial screening phase

**Figure 1:**
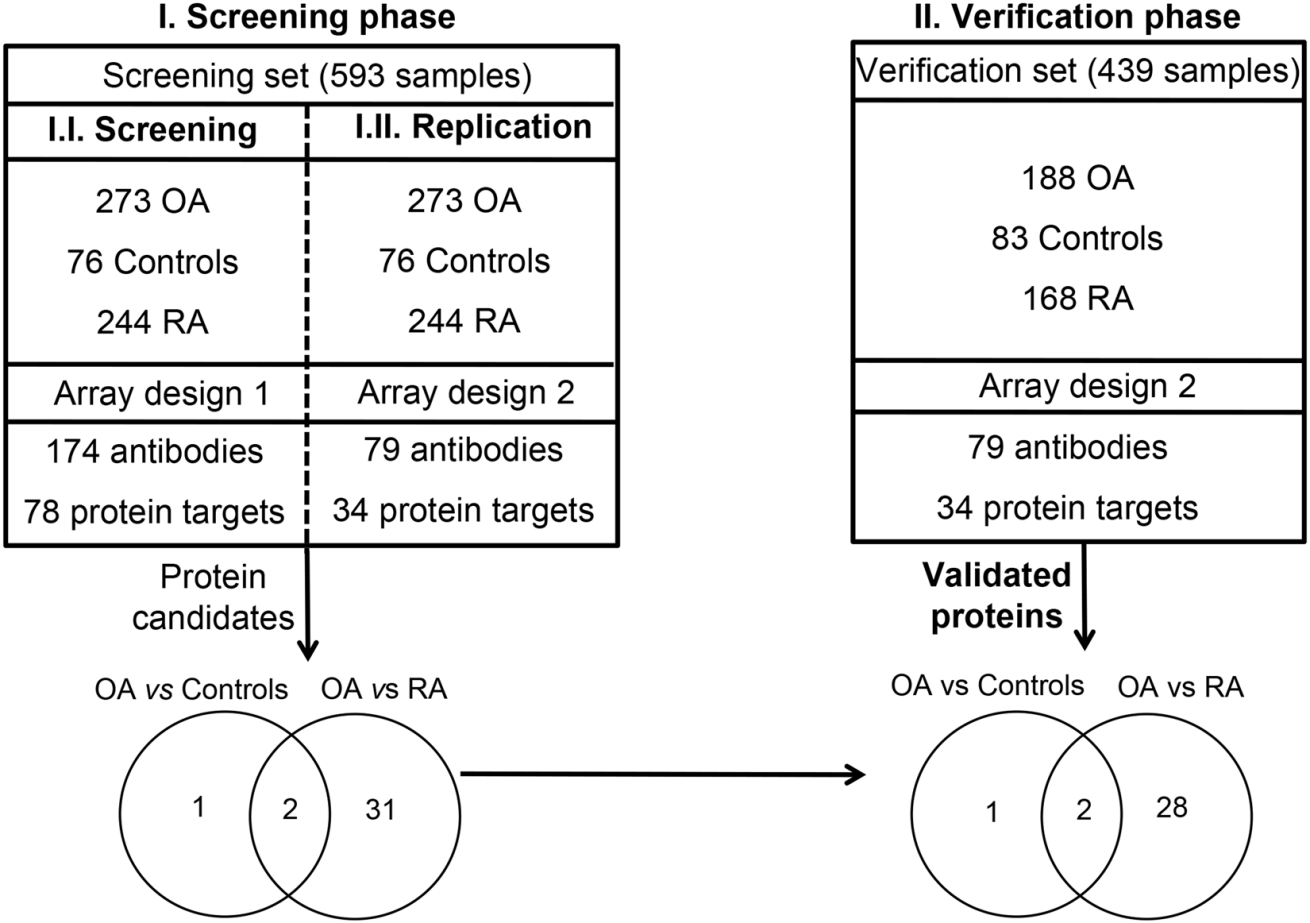
Schematic overview of the study. A screening phase was performed using a set of 593 serum samples and a protein array composed of 174 antibodies targeting 78 different proteins (Array 1, Phase I.I.). The levels of three proteins were found significantly (P < 0.05) different between osteoarthritis (OA) patients and control individuals, and 33 proteins differed between OA and rheumatoid arthritis (RA). A more focused array (Array 2) was then built targeting these 34 different proteins with 79 antibodies, and the results were replicated in the same sample set (Phase I.II.). Finally, a verification phase (II) was carried out using this second array on an independent set of 439 serum samples. In this phase, the three biomarker candidates separating OA and controls were verified, as well as 30 of the 33 proteins that were found with altered levels between OA and RA patients.

**Figure 2:**
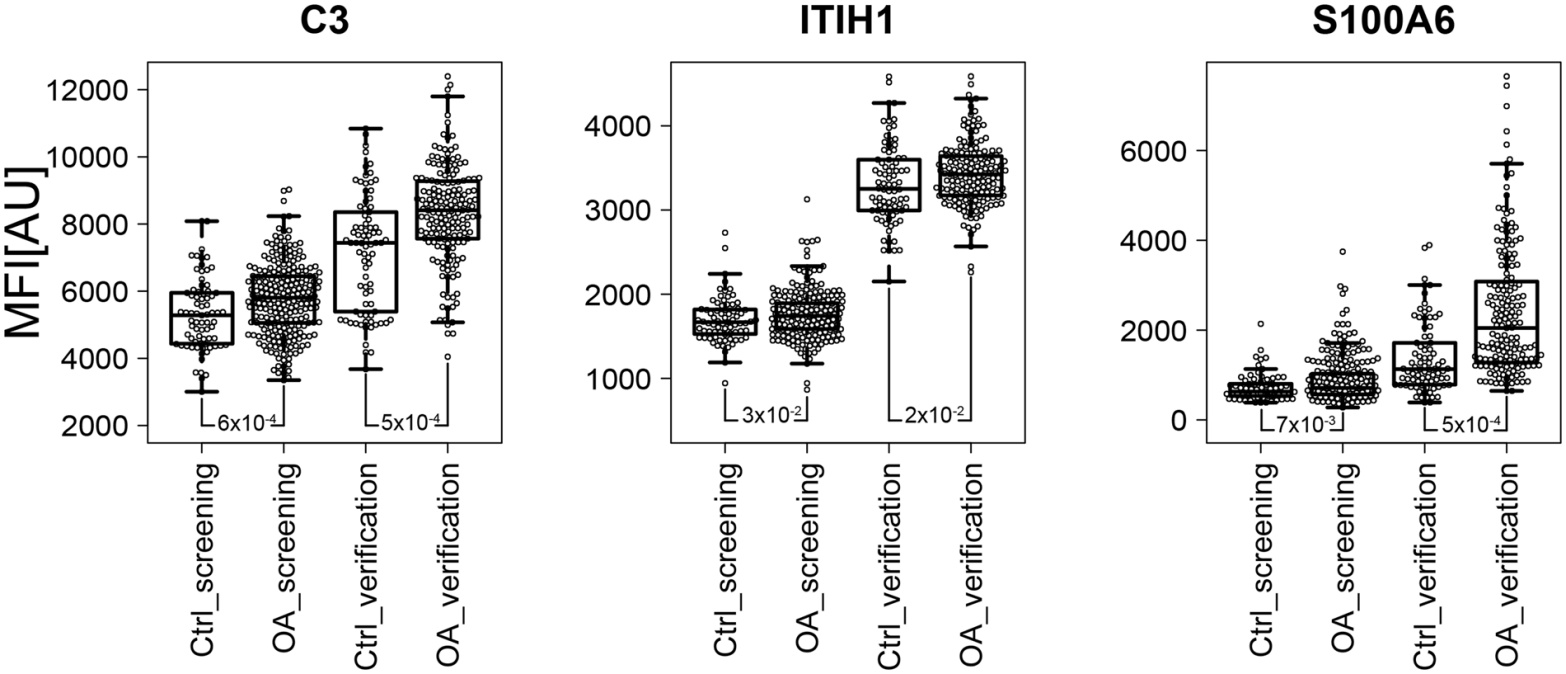
Identification of concordant protein profiles separating osteoarthritis patients from control groups in the screening and verification sample sets analysed separately. Box-plots illustrate the profiles for the three proteins concordantly revealing significant differences (P < 0.05) in the two sample sets analysed in this study for group comparisons between OA patients and controls. For each sample group, the box-and-whisker plot represents MFI values within lower and upper quantile (box), the median (horizontal line within box), percentiles of 5% and 95% (whiskers) and outliers (dots).

An overview of the strategy followed in this work for the large-scale proteomic analysis of sera is illustrated in Fig. 1. In the screening phase, we analysed a sample set composed of 273 OA, 76 controls and 244 RA subjects using a suspension bead array composed of 174 different antibodies targeting 78 different proteins (Array 1, Supplementary Table S1). Three proteins displayed levels significantly (P < 0.05) different between OA patients and healthy controls (Fig. 2), whereas 33 differed between OA and RA patients (Fig. 1). Among these, two proteins identified as distinguishing between OA and controls were also quantitatively different between OA patients compared to RA patients (Fig. 1). The results of this screening phase narrowed the list of candidates to 34 different proteins. Therefore, a more focused array comprising a total of 79 antibodies targeting these 34 proteins (Array 2, Supplementary Table S2) was used to profile the same set of samples. All the results were confirmed using this new array in the screening set (Supplementary Table S3A and S3B), which demonstrated the robustness of the technology and the reliability of the data obtained.

### Verification phase

Following the screening phase, a verification study was performed to profile the panel of these 34 proteins (Array 2) in an independent set of serum samples from 188 OA patients and 83 control subjects, as well as 168 RA patients (Table 1). In accordance with the findings observed in the screening phase, the same three proteins identified in the screening analyses were found to display levels allowing to significantly (P < 0.05) distinguish between OA and healthy individuals (Figs 1 and 2). Additionally, 30 proteins out of the 33 detected in the screening were verified as differing between OA and RA patients, being two of these 30 also modulated between OAs and Controls (Fig. 1, Supplementary Table S4A and S4B).

**Table 1:** Clinical characteristics of the patients and control subjects used in the study.

Screening set	Control	OA = 273			RA
		K/L = 2	K/L = 3	K/L = 4	
Number of subjects	76	110	106	57	244
Female (%)	55	74	86	88	75
BMI, kg/m^2^ (mean ± SD)	29 ± 6	31 ± 5	32 ± 6	32 ± 5	27 ± 5
Age, years (mean ± SD)	63 ± 9	66 ± 8	69 ± 9	69 ± 10	55 ± 14
**Verification set**	**Control**	**OA = 188**			**RA**
		**K/L = 2**	**K/L = 3**	**K/L = 4**	
Number of subjects	83	38	27	123	168
Female, %	59	76	78	78	74
BMI, kg/m^2^ (mean ± SD)	25 ± 4	31 ± 5	31 ± 4	32 ± 5	27 ± 5
Age, years (mean ± SD)	54 ± 19	67 ± 11	71 ± 7	71 ± 8	58 ± 12

BMI, indicates body mass index; K/L, Kellgren-Lawrence score and SD, standard deviation.

### Protein profiles for OA diagnosis

The screening and verification phases concordantly allowed the identification of three antibodies targeting three different proteins that revealed significant differences in abundance (P < 0.05) between radiographic knee OA patients and control individuals. These three antibodies were generated towards complement 3 (C3), inter-alpha trypsin inhibitor heavy chain 1 (ITIH1) and S100 calcium binding protein A6 regulator (S100A6). All of these three proteins showed higher levels in serum from OA patients when compared to the control group (Fig. 2).

**Figure 3:**
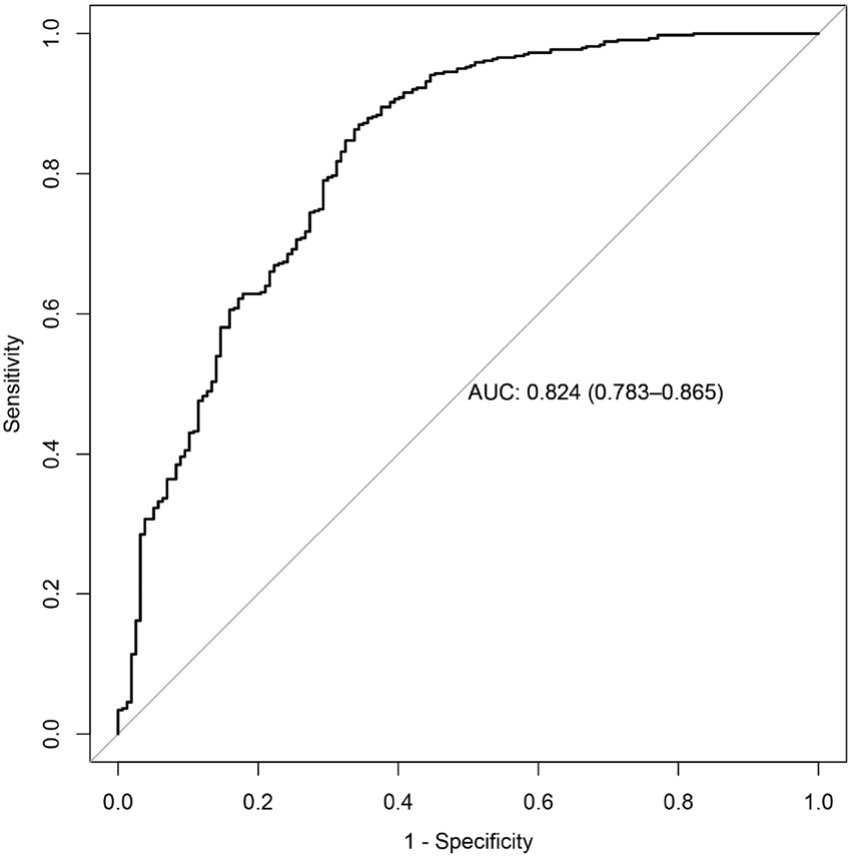
ROC curve representing the classification power of a panel composed of C3, ITIH1 and S100A6 profiles to discriminate between all OA patients and healthy control individuals analysed in this study.

The classification power of the identified and concordant protein profiles was visualized by a receiver operator characteristic (ROC) curve in the two sample sets combined. As shown in Fig. 3, this protein panel had an area under the curve (AUC) >0.82 for the classification between all OA patients and controls used in this study.

### Proteins associated with radiographic severity

**Figure 4:**
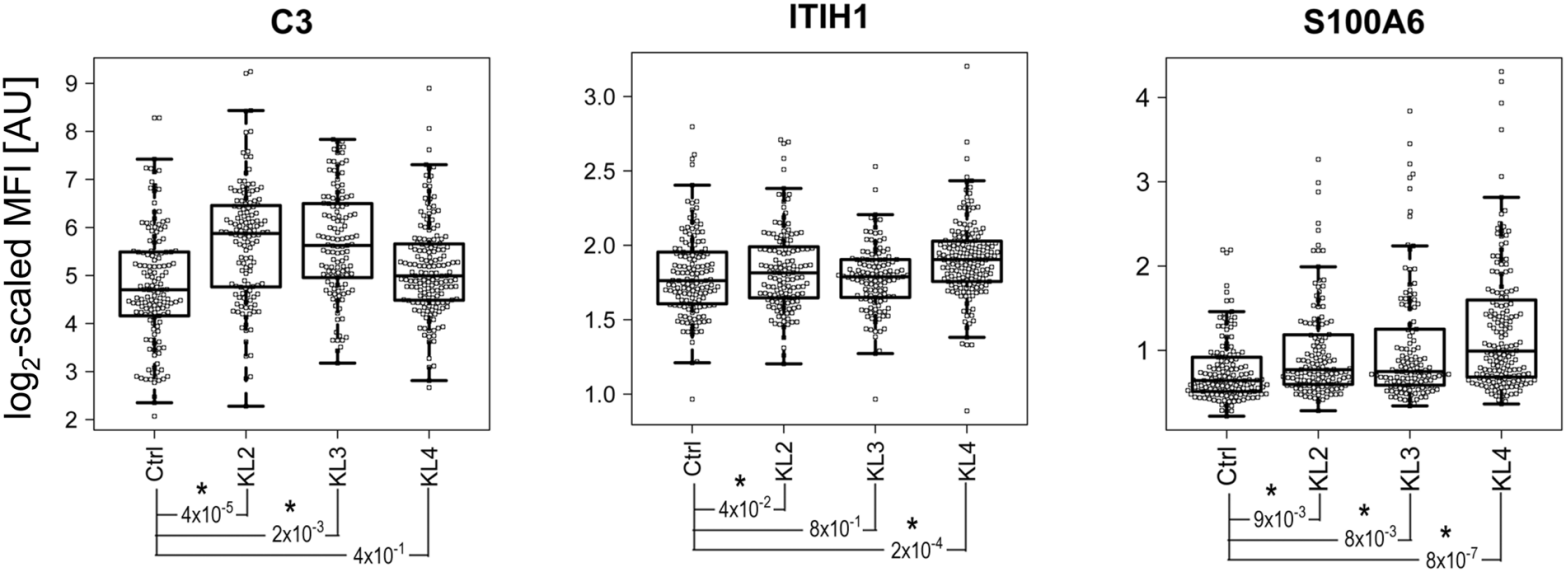
Box-plots showing the differential profiles of the three proteins between healthy controls and the different OA K/L groups from the two samples sets (screening and verification sets) combined. For each sample group, the box-and-whisker plot represents MFI values within lower and upper quantile (box), the median (horizontal line within box), percentiles of 5% and 95% (whiskers) and outliers (dots). Comparisons indicated with an * were statistically significant (P < 0.05).

After the identification of the protein panel concordantly distinguishing between OA patients and healthy controls in the two sample cohorts analysed separately (Fig. 2), both sample sets were combined to compare the profiles of C3, ITIH1 and S100A6 between the different OA K/L scored groups and healthy controls used in this study. A normalization step was followed to enable the combination of data from the two cohorts (see Methods), which was needed to provide a more homogeneous range of K/L grades for the comparisons. As shown in Fig. 4, patients with K/L = 2 showed significantly higher serum levels of C3, ITIH1 and S100A6 compared to controls. The same profiles were observed in the comparison between K/L = 3 and controls for C3 and S1006. Interestingly, significant differences were found already in OA K/L = 2 compared to healthy controls for all the three proteins. Only S100A6 showed higher levels in all K/L categories (K/L = 2, K/L = 3 and K/L = 4) compared to healthy controls (Fig. 4).

### Proteins distinguishing OA and RA patients

**Figure 5:**
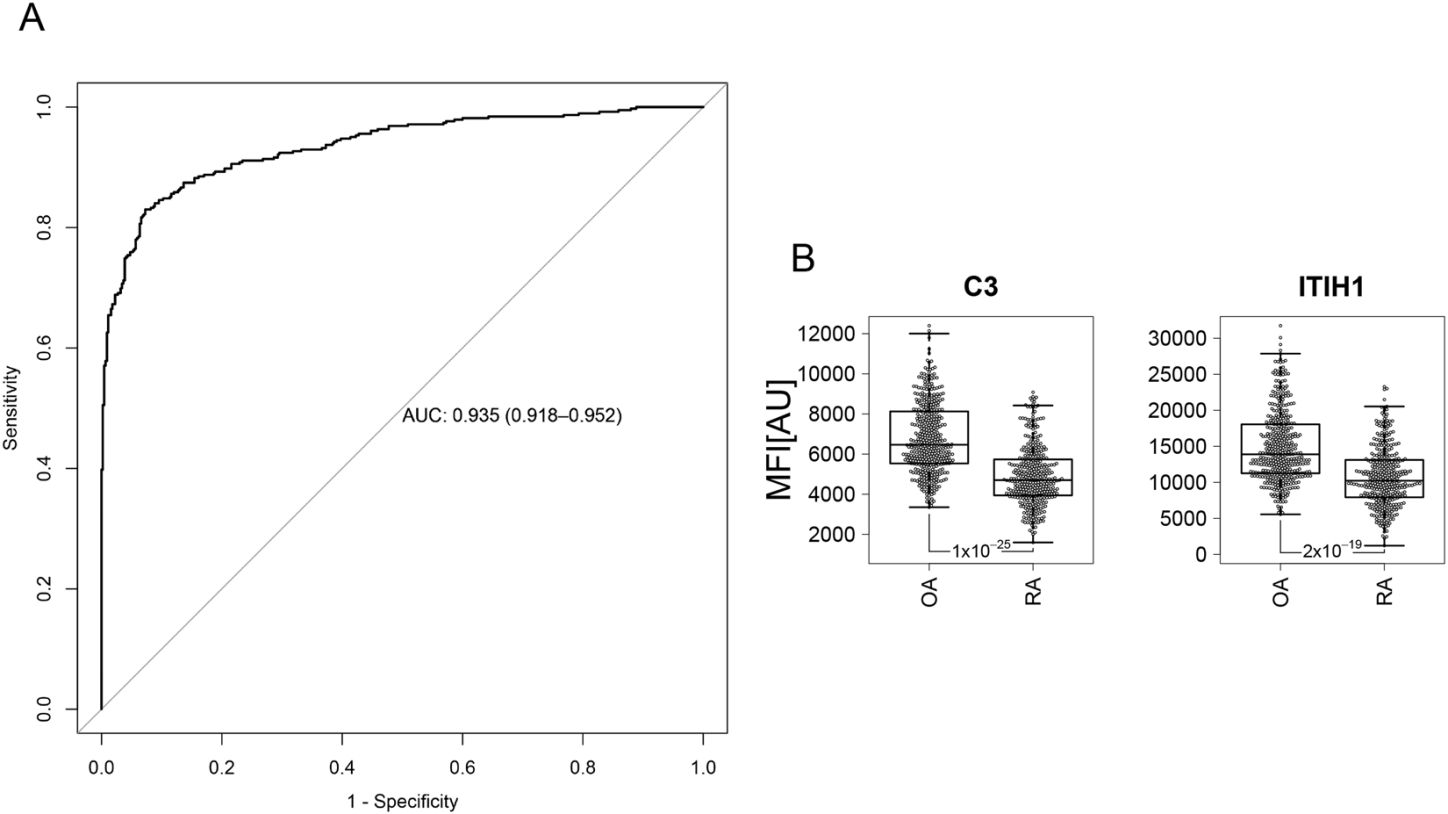
(**A**) ROC curve demonstrating the classification power of the panel of 30 proteins identified in this study for the classification between all OA patients and RA individuals included in the study. (**B**) Box-plots showing the two proteins revealing significant differences between OA patients and controls, which also revealed significant differences (P < 0.05) between OA and RA patients. For each sample group, the box-and-whisker plot represents MFI values within lower and upper quantile (box), the median (horizontal line within box), percentiles of 5% and 95% (whiskers) and outliers (dots).

The analysis of the screening and verification phases also allowed the detection of 30 proteins that significantly (P < 0.05) and concordantly differed between OA and RA patients in the two sample sets (Supplementary Table S4B). Among these 30 proteins, 18 were increased and 12 were decreased in OA patients compared to RA individuals. The classification performance of the identified and concordant protein profiles was visualized by a receiver operator characteristic (ROC) curve. The protein panel composed of these 30 proteins showed an area under the curve (AUC) >0.93 for classification between all OA and RA patients used in this study (Fig. 5A). The predicted biological roles of these proteins are represented in Supplementary Table S5, showing that 30% of them are related to inflammatory processes, 20% associated to bone remodelling, 20% involved in extracellular matrix (ECM) stability, 14% related to lipid metabolism and 16% implicated in other biological functions such as cell proliferation and transmembrane transport.

Finally, among the 30 proteins differing between OA and RA patients, we found that the levels of C3 and ITIH1, the two proteins significantly elevated in OA compared to controls (Fig. 2), were also significantly increased in OA patients compared to RA individuals (Fig. 5B).

## Discussion

We have performed an extensive profiling of serum samples using two different suspension antibody bead arrays with the aim of identifying protein profiles that could be associated with OA. Serum samples from a total of 1,032 individuals were analysed to evaluate the levels of up to 78 different proteins. To our knowledge, this high-throughput technology has been applied for the first time for the analysis of serum sample collections of this size within OA.

Using this high throughput and multiplex affinity proteomic approach, we identified three proteins (C3, ITIH1 and S100A6), whose levels in serum were significantly increased between radiographic OA and control individuals. These three proteins are therefore likely to be potential biochemical markers for this disease.

To evaluate the specificity of the protein panel, we analyzed samples from another rheumatic disease (RA) along with OA and controls. Interestingly, among the significantly modulated proteins, we found that two proteins, C3 and ITIH1 were concordantly and significantly increased in OA compared to both healthy controls d RA patients. Complement components are expressed by normal chondrocytes and their production is increased in the presence of fragments of extracellular matrix components. It is known that in OA the catabolic processes compromise the integrity of the cartilage, and fragments of proteins released from ECM as fibromodulin, COMP and osteoadherin lead to the activation of C1q component to further activate the classical and alternative pathways of complement factors^19^.

Low-grade inflammation is a feature already described in the literature as a driving force in the pathogenesis of the OA^20, 21^ and it is known that the inflammatory complement system plays a central role within this process^22^. Complement activation has also emerged as a crucial factor in experimental OA progression^23^. Increased levels of complement C3 in serum from late OA patients compared to healthy donors were already identified in previous proteomic screenings^24^. Furthermore, one of its fragments (C3f) was also described as increased in early OA compared to normal individuals and RA patients^25^.

In the present work, we have found that protein C3 levels are higher in serum from OA compared to all control subjects (healthy and RA), being significant higher when less severe OA stages (K/L = 2) were compared to healthy controls but they decrease when OA progresses (K/L = 3 and K/L = 4). Our results obtained for serum are in agreement with what has been already observed in synovial fluids^22^.

Taken together, we may speculate that C3 appeared to be increased in OA compared to healthy controls, but it decreases when OA progresses because the activation of complement cascade could be the main driver of inflammation at the first stages of the OA but at end stages (K/L = 4), this inflammatory pathway might switch to other biochemical pathways more associated to advanced OA and chronic arthritis such as RA.

An interesting data to support this hypothesis would be that in this work we also observed that levels of C3, together with other complement proteins, were significantly increased in OA patients compared to RA (Supplementary Table S5), which is a rheumatic disease more characterized by chronic and systemic inflammation than OA.

Therefore, using an antibody-based approach, our result confirms previous data described in the literature and point out the potential of C3 evaluation as an early marker of radiographic knee OA.

We also found that the levels of inter-alpha-trypsin inhibitor heavy chain 1 (ITIH1) were higher in OA patients compared to healthy controls and RA individuals. This protein also showed higher levels in all OA K/L grades compared to controls, although this was only statistically significant in K/L = 2 and K/L = 4 scores. It is known that ITIH1 is synthesized by chondrocytes and binds to hyaluronic acid and other extracellular matrix components^26, 27^ providing stability to the cartilage. This protein was found at higher levels in synovial fluids from OA patients compared to RA^28^, and this trend has been also detected in serum in the present work (Fig. 4). Therefore, our results show for the first time evidence in serum of the role of ITIH1 in OA and its potential value as a molecular signature of this disease.

Besides the additional evidence of the role of complement activation and ITIH1 in OA, our study also provides new insights into other pathogenic mechanisms of this disease. The identification of significantly increased levels of S100A6 (or calcyclin) in all K/L groups of OA serum samples compared to healthy controls points to a role of this protein in the OA process. Although this calcium-binding protein is known to be expressed in OA cartilage^29^ and there is an evidence of its expression in chondrocytes (data shown in HPA database, www.proteinatlas.org), its role in OA cartilage has so far not been described. It has been suggested that S100A6 could be involved in cell survival by interaction with advanced glycation end products (RAGE), consequent formation of reactive oxygen species (ROS), activation of ERK pathway and changes in NF-κB transcriptional activity, as well as promoting catabolic process in the cartilage^30, 31^. S100A6 was also described to enhance osteoblast proliferation and bone remodelling, however the underlying mechanisms are still unclear^32^. Therefore, our results suggest a potential value of this protein for diagnosis of OA, and underline the need of further functional studies to elucidate the specific role of S100A6 in OA.

In conclusion, we present for the first time an affinity proteomic approach comparing serum protein levels of OA, control and RA individuals in a total of 1,032 samples. Among the 78 different investigated proteins, targeted with 174 antibodies, we identified three proteins: Levels of C3, ITIH1 and S100A6 significantly differed between OA patients and healthy individuals, with the potential to be of additive value for the diagnosis and monitoring of OA. Interestingly, the serum levels of two of these proteins, C3 and ITIH1, differed also among OA and RA patients, which suggest C3 and ITIH1 are proteins specifically increased in OA. Taken together, upon further validations in independent sample collections, these findings help to a better understanding of OA pathology and provide a novel insight into the OA biomarker field.

## Methods

### Ethic statement

All methods were conducted according to the Declaration of Helsinki, which establish the regulations and guidelines for research project execution for human health. The research protocol was approved by the local Ethics Committee (Comité Ético de Galicia, Galicia, Spain). An informed written consent was obtained from all participants. The cohorts of patients included in this project were selected from the collections of samples already available and characterized at the Biobank of INIBIC, Collection of samples for research in Rheumatic Diseases (Cod. RNB C.0000424).

### Patients and controls

All individuals analysed in this study are included in specific cohorts localized in the Rheumatology Service of Hospital Universitario of A Coruña. All these individuals came at the Rheumatology Service of the Hospital to perform a regular clinical visit. The OA participants (n = 461) were diagnosed according to the American College of Rheumatology (ACR) criteria^33^, which exclude a disease of autoimmune etiology. and knee radiographies were classified using the Kellgren-Lawrence (K/L) score^34^. All patients with knee OA who tested positive for the autoantibodies rheumatoid factor, anti-nuclear (ANA) and anti-citrullinated antibodies (anti-CCP), were excluded from the study. Individuals were classified as controls (n = 159) based on following inclusion criteria: not autoimmune disease and non-radiographic knee OA. Additionally, a total of 412 RA patients fulfilling the ACR criteria^35^, were included in the study. The clinical data of the patients are summarized in Table 1.

### Samples

Blood samples from all patients and controls were collected after overnight fast in plain tubes containing a separation gel. The samples were allowed to stand for 20 min and then centrifuged at 2800 rpm for 10 min. The serum was aliquoted and stored at −80 °C until use.

### Antibody selection and bead array generation

Protein targets proposed to generate the antibody bead arrays were selected based on thorough mining of experimental evidence in the literature of rheumatic diseases^36–38^ and in previous in-house efforts in the field of osteoarthritis using mass spectrometry analyses^24, 28, 39^. The antibody set was finally designed according to the antibody availability within the Human Protein Atlas (HPA)^40^. A total of 174 protein microarray-validated polyclonal antibodies^41^ targeting 78 unique proteins were included in the arrays, selecting at least one antibody for each target protein (Supplementary Table S1).

The bead arrays were created as previously described^42, 43^ by diluting 1.6 μg of each antibody into 100 μL of antibody dilution buffer. All antibodies were immobilized onto color-coded magnetic beads (MagPlex-C, Luminex Corp.) with each bead identity corresponding to a unique antibody. The coupling of each antibody on the beads was confirmed via R-phycoerythrin-conjugated donkey anti rabbit-IgG antibody (Jackson ImmunoResearch).

### Serum profiling

The procedure for serum profiling was performed as described previously^43, 44^. Briefly, 3 μL of each sample were diluted 1:10 in phosphate-buffered saline (PBS), and randomized in 96-well plates. Then, the protein content was directly labelled with biotin (Life Technologies). Samples were further diluted 1:50 in an assay buffer, heated for 30 min at 56 degrees Celsius for 30 min, combined into a 384-well microtiter plate, and incubated with the bead array at room temperature on a shaker overnight. Unbound proteins were removed by washing and proteins captured on the beads were detected through a R-phycoerythrin-conjugated streptavidin (Invitrogen). Results from the FlexMap3D instrument (Luminex Corp.) were reported per bead identity as median fluorescence intensities (MFI).

### Study setup

An overview of the study design is illustrated in Fig. 1. A set of 593 samples (denoted as screening set) containing 273 OA patients, 76 control subjects and 244 RA patients was first analyzed (screening phase) using a panel of 174 antibodies immobilized on bead arrays, targeting 78 unique proteins (Array 1, Supplementary Table S1). In total, 34 different proteins showed altered levels in comparisons across the sample groups in this first screening. Secondly, a smaller panel comprising 79 antibodies targeting these 34 proteins (Array 2, Supplementary Table S2) was used to replicate the analysis on the same screening set, and then in a third step to verify the protein profiles identified in this screening phase using a new set of 439 samples (denoted as verification set), which was composed of 188 OA, 83 controls and 168 RA individuals.

### Statistical analysis

Data were processed and visualized in R. MFI values were normalized in each 384-plate by probabilistic quotient normalization (PQN) as accounting for any potential sample dilution effects^45^. In addition, potential batch effects were adjusted using the ComBat function included in the ‘sva’ R package. The outliers that were identified by robust principal component analysis (rPCA, R package “rrcov”) were excluded from further analysis.

The technical variation was assessed by calculating the coefficient of variation (C.V.), which was lower than 20% based on replicates of pooled samples distributed across all the plates (Supplementary Figure S1).

For biological interpretation, a linear regression analysis adjusting for sex, age and body mass index (BMI) was applied as a statistical test in order to identify differences in protein profiles between the compared groups (Figs 2 and 5B). Proteins were denoted significantly different between groups if the antibodies targeting each specific protein revealed unadjusted P < 0.05 both in screening and verification phases analysed separately.

We employed logistic regression to evaluate the classification power of the significant proteins concordantly distinguishing OA and controls (Fig. 3), as well as between OA and RA patients in the combination of the two sample sets (Fig. 5A), where ten-fold cross-validation method was selected as cross-validation option. The ROC curves were generated using the R package “pROC”.

For the K/L analysis, the two cohorts were combined to obtain a better range of K/L grades. The differences between both cohorts were minimised by dividing all MFI values in each cohort by the overall median MFI of the corresponding cohort. Before combining the two cohorts, a log2-scaling was performed to approximate the distribution of different antibodies in each cohort to the normal and, thus, to make them more alike. Finally, a linear regression analysis adjusting for sex, age and body mass index (BMI) was applied as a statistical test in order to identify differences in protein profiles between the different K/L grades and the controls (Fig. 4).

## Electronic supplementary material

Suplementary Figure 1

Supplementary Table 1

Supplementary Table2

Supplementary Table 3

Supplementary Table 4

Supplementary Table 5

## References

[1] Sherif El-Tawil EA, David Parker (2016). Position statement: the epidemiology, pathogenesis and risk factors of osteoarthritis of the knee. Journal of ISAKOS.

[2] Kraus VB, Blanco FJ, Englund M, Karsdal MA, Lohmander LS (2015). Call for standardized definitions of osteoarthritis and risk stratification for clinical trials and clinical use. Osteoarthritis Cartilage.

[3] Grazio S, Balen D (2009). [Obesity: risk factor and predictor of osteoarthritis]. Lijec Vjesn.

[4] Kraus VB (2015). OARSI Clinical Trials Recommendations: Soluble biomarker assessments in clinical trials in osteoarthritis. Osteoarthritis Cartilage.

[5] McConnell S, Kolopack P, Davis AM (2001). The Western Ontario and McMaster Universities Osteoarthritis Index (WOMAC): a review of its utility and measurement properties. Arthritis Rheum.

[6] Altman RD, Gold GE (2007). Atlas of individual radiographic features in osteoarthritis, revised. Osteoarthritis Cartilage.

[7] Eckstein F, Cicuttini F, Raynauld JP, Waterton JC, Peterfy C (2006). Magnetic resonance imaging (MRI) of articular cartilage in knee osteoarthritis (OA): morphological assessment. Osteoarthritis Cartilage.

[8] Guermazi A, Hayashi D, Roemer FW, Felson DT (2013). Osteoarthritis: a review of strengths and weaknesses of different imaging options. Rheum Dis Clin North Am.

[9] Finan PH (2013). Discordance between pain and radiographic severity in knee osteoarthritis: findings from quantitative sensory testing of central sensitization. Arthritis Rheum.

[10] Felson DT, Niu J, Guermazi A, Sack B, Aliabadi P (2011). Defining radiographic incidence and progression of knee osteoarthritis: suggested modifications of the Kellgren and Lawrence scale. Ann Rheum Dis.

[11] Ding C, Zhang Y, Hunter D (2013). Use of imaging techniques to predict progression in osteoarthritis. Curr Opin Rheumatol.

[12] Golightly YM (2011). Biomarkers of incident radiographic knee osteoarthritis: do they vary by chronic knee symptoms?. Arthritis Rheum.

[13] Rousseau J, Garnero P (2012). Biological markers in osteoarthritis. Bone.

[14] Lotz M (2014). Republished: Value of biomarkers in osteoarthritis: current status and perspectives. Postgrad Med J.

[15] Kraus VB (2011). Application of biomarkers in the development of drugs intended for the treatment of osteoarthritis. Osteoarthritis Cartilage.

[16] van Spil WE, DeGroot J, Lems WF, Oostveen JC, Lafeber FP (2010). Serum and urinary biochemical markers for knee and hip-osteoarthritis: a systematic review applying the consensus BIPED criteria. Osteoarthritis Cartilage.

[17] Hunter DJ (2010). A pathway and approach to biomarker validation and qualification for osteoarthritis clinical trials. Curr Drug Targets.

[18] Ayoglu B (2011). Systematic antibody and antigen-based proteomic profiling with microarrays. Expert Rev Mol Diagn.

[19] Sjoberg A, Onnerfjord P, Morgelin M, Heinegard D, Blom AM (2005). The extracellular matrix and inflammation: fibromodulin activates the classical pathway of complement by directly binding C1q. J Biol Chem.

[20] Kapoor M, Martel-Pelletier J, Lajeunesse D, Pelletier JP, Fahmi H (2011). Role of proinflammatory cytokines in the pathophysiology of osteoarthritis. Nat Rev Rheumatol.

[21] Loeser RF, Goldring SR, Scanzello CR, Goldring MB (2012). Osteoarthritis: a disease of the joint as an organ. Arthritis Rheum.

[22] Wang Q (2011). Identification of a central role for complement in osteoarthritis. Nat Med.

[23] Liu-Bryan R, Terkeltaub R (2015). Emerging regulators of the inflammatory process in osteoarthritis. Nat Rev Rheumatol.

[24] Fernandez-Puente P (2011). Identification of a panel of novel serum osteoarthritis biomarkers. Journal of proteome research.

[25] de Seny D (2011). Discovery and biochemical characterisation of four novel biomarkers for osteoarthritis. Ann Rheum Dis.

[26] Yoshihara Y (2008). Superficial zone chondrocytes in normal and osteoarthritic human articular cartilages synthesize novel truncated forms of inter-alpha-trypsin inhibitor heavy chains which are attached to a chondroitin sulfate proteoglycan other than bikunin. Osteoarthritis Cartilage.

[27] Zhao M (1995). Evidence for the covalent binding of SHAP, heavy chains of inter-alpha-trypsin inhibitor, to hyaluronan. J Biol Chem.

[28] Mateos J (2012). Differential protein profiling of synovial fluid from rheumatoid arthritis and osteoarthritis patients using LC-MALDI TOF/TOF. J Proteomics.

[29] Zreiqat H (2010). S100A8 and S100A9 in experimental osteoarthritis. Arthritis research & therapy.

[30] Donato R (2013). Functions of S100 proteins. Curr Mol Med.

[31] Leclerc E, Fritz G, Weibel M, Heizmann CW, Galichet A (2007). S100B and S100A6 differentially modulate cell survival by interacting with distinct RAGE (receptor for advanced glycation end products) immunoglobulin domains. J Biol Chem.

[32] Zhou Z, Xiong WC (2011). RAGE and its ligands in bone metabolism. Front Biosci (Schol Ed).

[33] Altman R (1986). Development of criteria for the classification and reporting of osteoarthritis. Classification of osteoarthritis of the knee. Diagnostic and Therapeutic Criteria Committee of the American Rheumatism Association. Arthritis Rheum.

[34] Kellgren JH, Lawrence JS (1957). Radiological assessment of osteo-arthrosis. Ann Rheum Dis.

[35] Aletaha D (2010). 2010 Rheumatoid arthritis classification criteria: an American College of Rheumatology/European League Against Rheumatism collaborative initiative. Arthritis Rheum.

[36] Mc Ardle A, Flatley B, Pennington SR, FitzGerald O (2015). Early biomarkers of joint damage in rheumatoid and psoriatic arthritis. Arthritis research & therapy.

[37] Bay-Jensen AC (2016). Osteoarthritis year in review 2015: soluble biomarkers and the BIPED criteria. Osteoarthritis Cartilage.

[38] Mobasheri A, Bay-Jensen AC, van Spil WE, Larkin J, Levesque MC (2017). Osteoarthritis Year in Review 2016: biomarkers (biochemical markers). Osteoarthritis Cartilage.

[39] Lourido L (2014). Quantitative proteomic profiling of human articular cartilage degradation in osteoarthritis. Journal of proteome research.

[40] Uhlen M (2010). Towards a knowledge-based Human Protein Atlas. Nat Biotechnol.

[41] Sjoberg R (2012). Validation of affinity reagents using antigen microarrays. N Biotechnol.

[42] Drobin K, Nilsson P, Schwenk JM (2013). Highly multiplexed antibody suspension bead arrays for plasma protein profiling. Methods Mol Biol.

[43] Schwenk JM, Nilsson P (2011). Antibody suspension bead arrays. Methods Mol Biol.

[44] Schwenk JM, Gry M, Rimini R, Uhlen M, Nilsson P (2008). Antibody suspension bead arrays within serum proteomics. Journal of proteome research.

[45] Dieterle F, Ross A, Schlotterbeck G, Senn H (2006). Probabilistic quotient normalization as robust method to account for dilution of complex biological mixtures. Application in 1H NMR metabonomics. Anal Chem.

