# Supplementary material for: Discovery of circulating proteins associated to knee radiographic osteoarthritis
Source: Sci Rep. 2017 Mar 9;7:137. doi: 10.1038/s41598-017-00195-8 (PMC5427840; doi:10.1038/s41598-017-00195-8)
Supplement: Supplementary file 1 — Suplementary Figure 1 [file 41598_2017_195_MOESM1_ESM.pdf]

Discovery of circulating proteins associated to knee radiographic osteoarthritis

Lucía Lourido, Burcu Ayoglu, Juan Fernández-Tajes, Natividad Oreiro, Frauke Henjes, Cecilia Hellström, Jochen M. Schwenk, Cristina Ruiz-Romero, Peter Nilsson, Francisco J. Blanco

Supplementary information

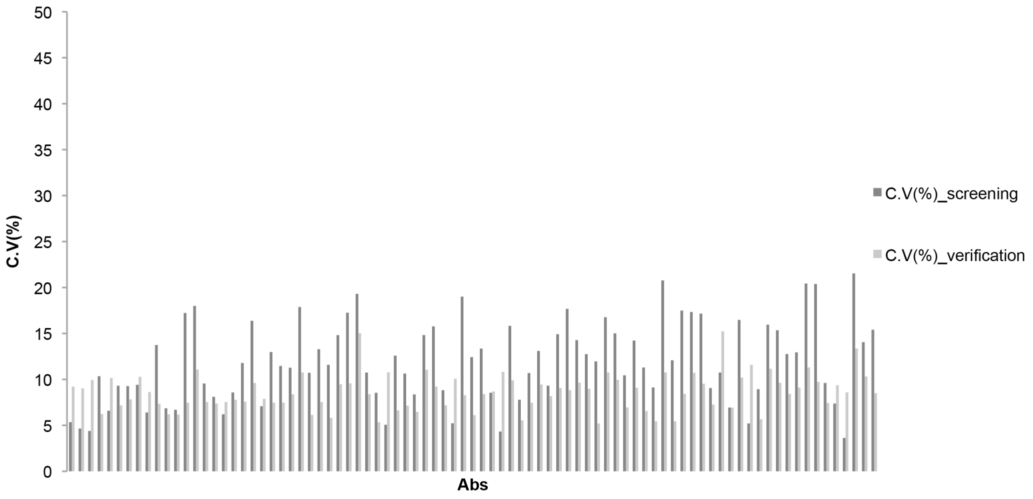

Supplementary Figure S1. Distribution of intra-assay variance in the screening and verification assays. The plot shows the coefficient of variation (C.V) in the technical replicates. The technical quality of the assays was assessed by the intra-assay C.V using MFI values over the replicates pooled serum samples. The C.V of all antibodies concordantly used in the screening and verification sample sets was lower than 20%.
